# Supplementary material for: Sea star wasting syndrome reaches the high Antarctic: Two recent outbreaks in McMurdo Sound
Source: PLoS One. 2023 Jul 27;18(7):e0282550. doi: 10.1371/journal.pone.0282550 (PMC10374074; doi:10.1371/journal.pone.0282550)
Supplement: Attachment — Submitted filename: Response to Reviewers.docx [file pone.0282550.s002.docx]

PONE-D-23-04532

Sea Star Wasting Syndrome Reaches the High Antarctic: Two Recent Outbreaks in McMurdo Sound

PLOS ONE

Dear Dr. Grun,

Thank you for the review and editorial comments on the first version of the MS. We have carefully considered all the suggestions for minor revisions and in most cases, we have made the recommended changes. We appreciate the positive comments of the reviewer and feel that the MS is improved.

In the Response to Reviewers, we address each editorial and reviewer comment individually.

Thank you and best wishes,

Amy Moran and coauthors

School of Life Sciences

University of Hawai'i at Mānoa

Response to Reviewers

Editorial and reviewer comments in *italics*

*1. Please ensure that your manuscript meets PLOS ONE's style requirements, including those for file naming. The PLOS ONE style templates can be found at 
https://journals.plos.org/plosone/s/file?id=wjVg/PLOSOne_formatting_sample_main_body.pdf and* 
[*https://journals.plos.org/plosone/s/file?id=ba62/PLOSOne_formatting_sample_title_authors_affiliations.pdf*](https://journals.plos.org/plosone/s/file?id=ba62/PLOSOne_formatting_sample_title_authors_affiliations.pdf)

We have followed these style requirements to the best of our ability.

*2. In your Methods section, please provide additional information regarding the permits you obtained for the work. Please ensure you have included the full name of the authority that approved the field site access and, if no permits were required, a brief statement explaining why.*

We have added the following text to the MS (lines 86-89 in the marked-up manuscript): “No organisms were taken during this project. No permits were required for site access because the study sites were not within any Antarctic Specially Protected Areas (ASPA) or Antarctic Specially Managed Areas (ASMA) as defined by Annex V to the Environment Protocol, Agreed Measures for the Conservation of Antarctic Fauna and Flora, Antarctic Treaty Consultative Meeting.”

*3. Thank you for stating the following in the Acknowledgments Section of your manuscript: 
"Antarctic Service Contract diver R.G. Robbins was the first to see SSWS at the McMurdo Intake Jetty in 2019 and supplied data on diving history at McMurdo Station.  We thank both R.G. Robbins and S. Rupp for essential help with surveys at the McMurdo Intake Jetty in 2019 and 2020, as well as observations at other sites. R. Robbins performed all surveys at Cinder Cones in 2022. R. Robbins and S. Rupp made all observations at Granite Harbor, New Harbor, and Delbridge Seamount in 2022.We also thank McMurdo Station support staff for facilitating this research along with team members M.W. Aaron Toh, Graham Lobert, and L. Ardor Bellucci for assisting with the research. This work was funded by NSF-OPP-1745130 to ALM and NSF-OPP-2046800 to ART.":*

*We note that you have provided funding information that is not currently declared in your Funding Statement. However, funding information should not appear in the Acknowledgments section or other areas of your manuscript. We will only publish funding information present in the Funding Statement section of the online submission form. 
Please remove any funding-related text from the manuscript and let us know how you would like to update your Funding Statement. Currently, your Funding Statement reads as follows: 
"Funded by NSF-OPP-1745130 to ALM and NSF-OPP-2046800 to ART. United States National Science Foundation Office of Polar Programs, https://www.nsf.gov/div/index.jsp?div=OPP. The funders had no role in study design, data collection and analysis, decision to publish, or preparation of the manuscript."*

*Please include your amended statements within your cover letter; we will change the online submission form on your behalf*.

We have removed the funding-related text from the manuscript (strikethrough in lines 252-253, marked-up copy). No changes are needed to the Funding Statement.

*4. We note that Figure 1 in your submission contain [map/satellite] images which may be copyrighted. All PLOS content is published under the Creative Commons Attribution License (CC BY 4.0), which means that the manuscript, images, and Supporting Information files will be freely available online, and any third party is permitted to access, download, copy, distribute, and use these materials in any way, even commercially, with proper attribution. For these reasons, we cannot publish previously copyrighted maps or satellite images created using proprietary data, such as Google software (Google Maps, Street View, and Earth). For more information, see our copyright guidelines: http://journals.plos.org/plosone/s/licenses-and-copyright.
 
We require you to either (1) present written permission from the copyright holder to publish these figures specifically under the CC BY 4.0 license, or (2) remove the figures from your submission:
 
a. You may seek permission from the original copyright holder of Figure 1 to publish the content specifically under the CC BY 4.0 license.  
 
We recommend that you contact the original copyright holder with the Content Permission Form (http://journals.plos.org/plosone/s/file?id=7c09/content-permission-form.pdf) and the following text: “I request permission for the open-access journal PLOS ONE to publish XXX under the Creative Commons Attribution License (CCAL) CC BY 4.0 (http://creativecommons.org/licenses/by/4.0/). Please be aware that this license allows unrestricted use and distribution, even commercially, by third parties. Please reply and provide explicit written permission to publish XXX under a CC BY license and complete the attached form.”
 
Please upload the completed Content Permission Form or other proof of granted permissions as an "Other" file with your submission.
 
In the figure caption of the copyrighted figure, please include the following text: “Reprinted from [ref] under a CC BY license, with permission from [name of publisher], original copyright [original copyright year].”
 
b. If you are unable to obtain permission from the original copyright holder to publish these figures under the CC BY 4.0 license or if the copyright holder’s requirements are incompatible with the CC BY 4.0 license, please either i) remove the figure or ii) supply a replacement figure that complies with the CC BY 4.0 license. Please check copyright information on all replacement figures and update the figure caption with source information. If applicable, please specify in the figure caption text when a figure is similar but not identical to the original image and is therefore for illustrative purposes only.
The following resources for replacing copyrighted map figures may be helpful:
 
USGS National Map Viewer (public domain): http://viewer.nationalmap.gov/viewer/
The Gateway to Astronaut Photography of Earth (public domain): http://eol.jsc.nasa.gov/sseop/clickmap/
Maps at the CIA (public domain): https://www.cia.gov/library/publications/the-world-factbook/index.html and https://www.cia.gov/library/publications/cia-maps-publications/index.html
NASA Earth Observatory (public domain): http://earthobservatory.nasa.gov/
Landsat: http://landsat.visibleearth.nasa.gov/
USGS EROS (Earth Resources Observatory and Science (EROS) Center) (public domain): http://eros.usgs.gov/#
Natural Earth (public domain):* [*http://www.naturalearthdata.com/*](http://www.naturalearthdata.com/)

Figure 1 is compliant with the CC BY 4.0 license. The map image was obtained from Landsat, Image Mosaic of Antarctica, and is in the public domain: “Terms of Use: These images are in the public domain and can be used freely and without acknowledgement. However, credit to the Landsat Image Mosaic of Antarctica (LIMA) Project is greatly appreciated.”

https://developers.google.com/earth-engine/datasets/catalog/USGS_LIMA_SR#terms-of-use

We have amended the Figure 1 caption to read “The map used in Figure 1 is modified from the Landsat Image Mosaic of Antarctica (LIMA) Project and is in the public domain.” (lines 93-95, marked-up copy)

*5. We note that Figures 2 and 3 in your submission contain copyrighted images. All PLOS content is published under the Creative Commons Attribution License (CC BY 4.0), which means that the manuscript, images, and Supporting Information files will be freely available online, and any third party is permitted to access, download, copy, distribute, and use these materials in any way, even commercially, with proper attribution. For more information, see our copyright guidelines: http://journals.plos.org/plosone/s/licenses-and-copyright.
 
We require you to either (1) present written permission from the copyright holder to publish these figures specifically under the CC BY 4.0 license, or (2) remove the figures from your submission:*

*a. You may seek permission from the original copyright holder of Figures 2 and 3 to publish the content specifically under the CC BY 4.0 license. 
 
We recommend that you contact the original copyright holder with the Content Permission Form (http://journals.plos.org/plosone/s/file?id=7c09/content-permission-form.pdf) and the following text: “I request permission for the open-access journal PLOS ONE to publish XXX under the Creative Commons Attribution License (CCAL) CC BY 4.0 (http://creativecommons.org/licenses/by/4.0/). Please be aware that this license allows unrestricted use and distribution, even commercially, by third parties. Please reply and provide explicit written permission to publish XXX under a CC BY license and complete the attached form.”
 
Please upload the completed Content Permission Form or other proof of granted permissions as an "Other" file with your submission. 
 
In the figure caption of the copyrighted figure, please include the following text: “Reprinted from [ref] under a CC BY license, with permission from [name of publisher], original copyright [original copyright year].”
 
b. If you are unable to obtain permission from the original copyright holder to publish these figures under the CC BY 4.0 license or if the copyright holder’s requirements are incompatible with the CC BY 4.0 license, please either i) remove the figure or ii) supply a replacement figure that complies with the CC BY 4.0 license. Please check copyright information on all replacement figures and update the figure caption with source information. If applicable, please specify in the figure caption text when a figure is similar but not identical to the original image and is therefore for illustrative purposes only.*

For Figures 2 and 3, we have obtained and uploaded the signed CC BY 4.0 license from the copyright holder. We have amended both figure captions to read “Photographs by Rob Robbins, shared with permission under CC BY 4.0.” (lines 120 and 123-124, marked-up copy)

*6. Please include captions for your Supporting Information files at the end of your manuscript, and update any in-text citations to match accordingly. Please see our Supporting Information guidelines for more information: http://journals.plos.org/plosone/s/supporting-information*.

The caption for the Supporting Information (S1 Table) is at the end of the manuscript, after the references (lines 370-372, marked-up copy). We have amended it slightly to match the new text in the Results:

**S1 Table.** **Survey Metadata**. All count~~s~~, percentage, and density data ~~of~~ from surveys of *Odontaster validus* ~~from surveys~~ at the McMurdo Intake Jetty and Cinder Cones.

*Reviewers' comments:

Reviewer #1: This manuscript provides important information on the presence of SSE in a key Antarctic asteroid and support its publication. Overall, the manuscript is a straightforward read, although is a bit too descriptive*.

We appreciate these comments!

*I have suggestions that the authors should consider.

1. There should be some statistical analysis comparing data between years.*

We have added a chi-square test showing that the incidence of disease symptoms at the Intake Jetty was significantly lower in 2020 than in 2019.

The following text was added to the Methods (lines 113-118, marked-up copy):

“A chi-square test of independence was performed to compare the frequencies of asymptomatic animals (stage 0) to symptomatic animals (pooled stages 1-4) at the McMurdo Intake Jetty between 2019 and 2020. The same transect lines were surveyed twice in 2019, so to avoid potential double-counting of animals, for 2019 we only used data from the first sampling date on each side of the Jetty (10/17/19 for JS, 10/19/19 for JN). For both 2019 and 2020, data from JN and JS were pooled within year. All data are shown in S1 Table. The analysis was performed in JMP Pro 15.2.0 (SAS Institute Inc., 2019).”

The following text was added to the Results (lines 160-162, marked-up copy):

“A chi-square test showed that there was a significant relationship between disease frequency and year, χ^2^ (1, N = 909) = 64.24, p <0.0001. Symptomatic animals were significantly less frequent in 2020 compared to 2019.”

*2. L. 145, 179 mentions density. Densities are not provided and these data should be.*

All density data are in S1 Table. To clarify that the data are provided, we have amended the first line of the Results to read “All raw survey count data, percentages, and densities are provided in the supplementary information (S1 Table).” (line 148)

*Are there previous studies of the density of Odontaster in the region to compare with?*

This is an interesting suggestion, but the density of *Odontaster* is variable on a small spatial scale at these dive sites, and no previous data are available from our specific transect locations.

*3. For all of the %’s provide the sample size that this is based on.*

All sample sizes are already given in S1 Table. We feel that presenting count data in a table is much clearer and more readable then presenting the same data in the text, so we have chosen not to duplicate the table data in the text.

*4. What other sea stars are present? It will be good to name them and indicate that they were not infected.*

We agree this would be interesting to know this, but we do not have these data. We have changed the text to read “Similar to the event at Deception Island, symptoms of SSWS in McMurdo Sound were localized to particular dive sites and were not noted in any other species of asteroid besides *O. validus*. *O. validus* was by far the most common species at our sites, though, and other species were not surveyed in a systematic way. Therefore, SSWS in other species cannot be ruled out.” (lines 189-192, marked-up copy)

*5. There is a new issue of Biological Bulletin devoted to SSW – I suggest some of the papers in this issue should be consulted and cited as the most up to date.*

We could not find a recent issue of the The Biological Bulletin devoted to SSWS. We did find three papers in the December 2022 issue, which may be what the reviewer meant, but in our opinion these papers did not contain new data or observations that seemed relevant to this manuscript. Without more specific guidance from the reviewer, therefore, we are not sure how to change the MS in response to this comment.

*[NOTE: If reviewer comments were submitted as an attachment file, they will be attached to this email and accessible via the submission site. Please log into your account, locate the manuscript record, and check for the action link "View Attachments". If this link does not appear, there are no attachment files.]

While revising your submission, please upload your figure files to the Preflight Analysis and Conversion Engine (PACE) digital diagnostic tool,* [*https://pacev2.apexcovantage.com/*](https://pacev2.apexcovantage.com/)*. PACE helps ensure that figures meet PLOS requirements. To use PACE, you must first register as a user. Registration is free. Then, login and navigate to the UPLOAD tab, where you will find detailed instructions on how to use the tool. If you encounter any issues or have any questions when using PACE, please email PLOS at* [*figures@plos.org*](mailto:figures@plos.org)*. Please note that Supporting Information files do not need this step.*
